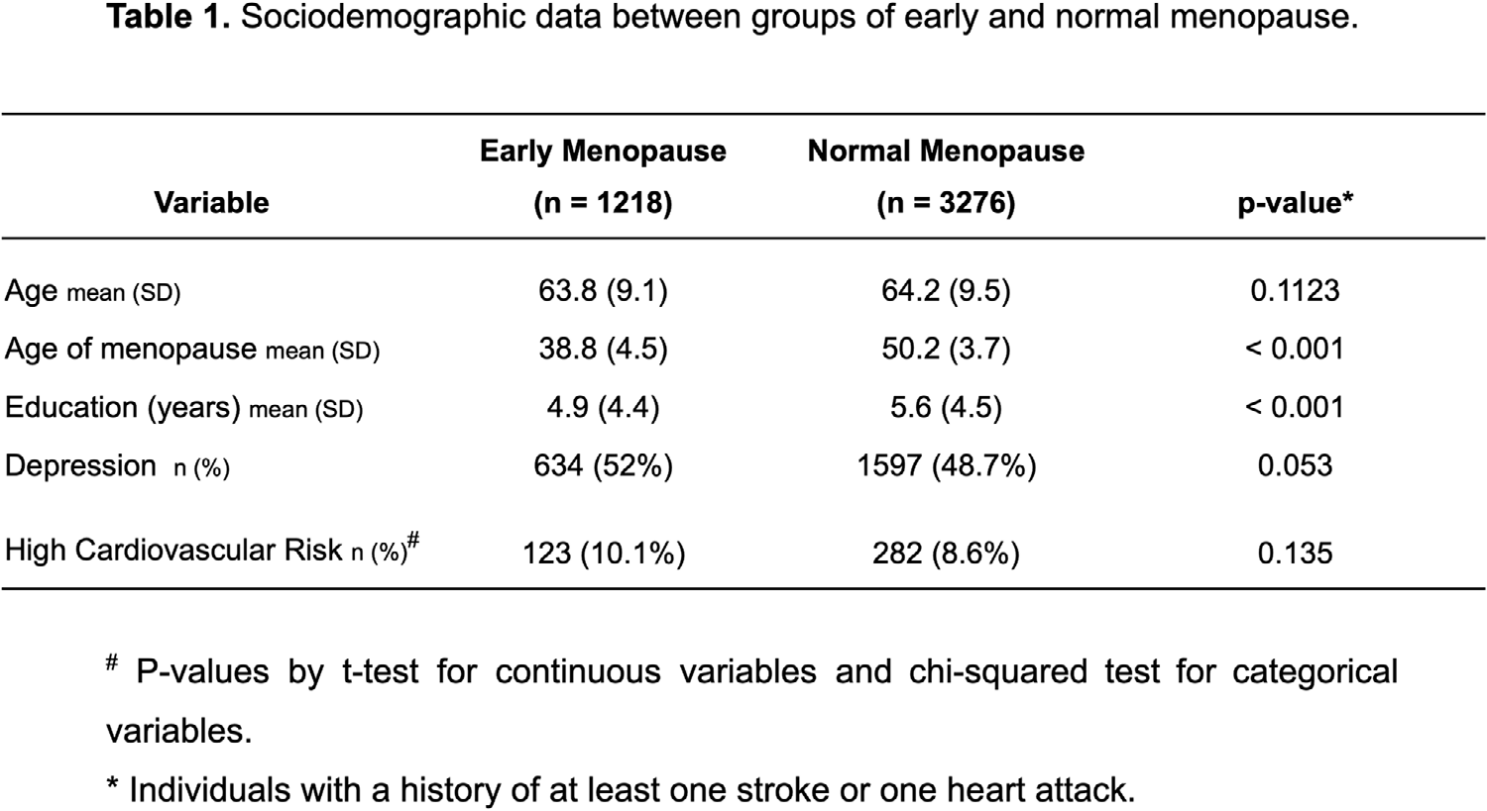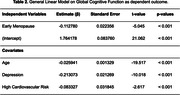# Early menopause and cognitive impairment: Insights from the ELSI‐Brazil Study

**DOI:** 10.1002/alz.093431

**Published:** 2025-01-03

**Authors:** Alvaro de Oliveira Franco, Vanessa Bielenfeldt Leotti, Eduardo R. Zimmer, Raphael Machado Castilhos

**Affiliations:** ^1^ Hospital de Clinicas de Porto Alegre, Porto Alegre, Rio Grande do Sul Brazil; ^2^ Universidade Federal do Rio Grande do Sul, Porto Alegre, Rio Grande do Sul Brazil; ^3^ Universidade Federal do Rio Grande do Sul, Porto Alegre Brazil

## Abstract

**Background:**

Dementia and cognitive impairment, primarily linked to aging, are increasingly recognized as multifactorial conditions. Emerging research has pointed to early menopause as another potential risk factor. However, its relationship with cognitive impairment needs further investigation, especially in low‐ and middle‐income countries. Therefore, this study aims to analyze the effect of early menopause on cognitive impairment in the Brazilian population.

**Methods:**

We analyzed data from the Brazilian Longitudinal Study of Aging (ELSI‐Brazil), a nationally representative study of individuals older than 50 years. In this analysis, we included only women (n = 4,494) and defined early menopause as when its age of onset occurred before 45 years. A Global Cognitive Score (GCS) was computed by standardizing Z scores yielded by the assessment of four cognitive subdomains (orientation, fluency, episodic memory, and semantic memory). Depression was assessed using the Epidemiological Scale‐Depression 8 (CES‐D8), with scores of 3 or higher indicating depression. High cardiovascular risk (HCR) was defined by a history of stroke or heart attack. We conducted a general linear model with GCS as the dependent variable, and early menopause, age, HCR, and depression as covariates. Statistical analyses were performed in the R environment, and significance was set at p < 0.05.

**Results:**

Of the 4,494 women included in the study, 1,218 (27.1%) had early menopause. Age between groups is not different, but the group with early menopause had fewer years of education than women with normal menopause (p < 0.001) (Table 1). Early menopause is significantly and independently associated with lower scores in GCS (β: ‐0.112, p <0.001), implying a lower performance in the cognitive assessment. Seemingly, depression (p < 0.001), age (p < 0.001), and high cardiovascular risk (p = 0.009) negatively impact GCS.

**Conclusions:**

Our study shows early menopause as an independent factor associated with cognitive impairment in a representative sample of the Brazilian population. Further research is warranted to elucidate the underlying mechanisms linking early menopause to cognitive impairment and to develop effective interventions that can optimize cognitive health in this vulnerable population.